# Divergent Expression Regulation of Gonad Development Genes in Medaka Shows Incomplete Conservation of the Downstream Regulatory Network of Vertebrate Sex Determination

**DOI:** 10.1093/molbev/mst130

**Published:** 2013-07-24

**Authors:** Amaury Herpin, Mateus C. Adolfi, Barbara Nicol, Maria Hinzmann, Cornelia Schmidt, Johanna Klughammer, Mareen Engel, Minoru Tanaka, Yann Guiguen, Manfred Schartl

**Affiliations:** ^1^University of Wuerzburg, Physiological Chemistry, Biocenter, Am Hubland, Wuerzburg, Germany; ^2^INRA, UR1037, LPGP, Fish Physiology and Genomics, Rennes, France; ^3^Laboratory of Molecular Genetics for Reproduction, National Institute for Basic Biology, Okazaki, Japan

**Keywords:** gene regulatory network evolution, divergent expression regulation, gonadal development, adaptive evolution

## Abstract

Genetic control of male or female gonad development displays between different groups of organisms a remarkable diversity of “master sex-determining genes” at the top of the genetic hierarchies, whereas downstream components surprisingly appear to be evolutionarily more conserved. Without much further studies, conservation of sequence has been equalized to conservation of function. We have used the medaka fish to investigate the generality of this paradigm. In medaka, the master male sex-determining gene is *dmrt1bY*, a highly conserved downstream regulator of sex determination in vertebrates. To understand its function in orchestrating the complex gene regulatory network, we have identified targets genes and regulated pathways of Dmrt1bY. Monitoring gene expression and interactions by transgenic fluorescent reporter fish lines, in vivo tissue-chromatin immunoprecipitation and in vitro gene regulation assays revealed concordance but also major discrepancies between mammals and medaka, notably amongst spatial, temporal expression patterns and regulations of the canonical Hedgehog and R-spondin/Wnt/Follistatin signaling pathways. Examination of Foxl2 protein distribution in the medaka ovary defined a new subpopulation of theca cells, where ovarian-type *aromatase* transcriptional regulation appears to be independent of Foxl2. In summary, these data show that the regulation of the downstream regulatory network of sex determination is less conserved than previously thought.

## Introduction

Sex determination, the decision whether the bipotential gonad anlage will become a testis or an ovary, is a complex and tightly controlled developmental process. The fate determination and cell differentiation programs are regulated and tuned by cascades or networks of genes. Comparative studies on sex determination cascades of different organisms revealed a remarkable diversity of “master sex-determining genes” at the top of the genetic hierarchies, whereas downstream components surprisingly appeared to be evolutionarily more conserved and tend to converge upon the regulation of common effectors. Hence, a comparative view on genetic sex determination mechanisms led to the paradigm that “masters change, slaves remain” (Graham et al. 2003). A well-known example illustrating this paradigm is the *SRY* gene, the master sex-determining gene of mammals, which has not been detected outside of the therian mammals. However, its subordinated genes (*SOX9*, *WT1*, *DMRT1*, *AMH*, *SF1*, *FOXL2*) or signaling pathways (TGF-beta, WNT4/beta-catenin, Hedgehog) have homologs in a much broader spectrum of species, including nonvertebrates, where they apparently are also involved in sex determination. These observations led to the emergence of the subversive stereotype that master sex-determining genes individually spark and orchestrate the irreversible action of uniform and integrated gender-specific pathways. Conservation at the bottom and diversity at the top could be convincingly explained by an evolutionary scenario in which these hierarchies evolve from common core downstream components that acquire new upstream regulators (Wilkins 2007). Although the global rule of sex determination evolution is intuitively appealing and well accepted, only the variety at the top is well supported by comparative experimental data (Graham et al. 2003; Haag and Doty 2005; Herpin and Schartl 2008). The downstream conservation is less studied and relies only on a few gene expression studies. We have used the medaka as a versatile model system to study gene regulatory interactions and their evolutionary conservation (Wittbrodt et al. 2002; Herpin and Schartl 2009).

In the medaka fish, which has XY-XX sex determination, *dmrt1bY*, the duplicated copy on the Y-chromosome of *dmrt1a*, was shown to be the dominant master regulator of male development (Matsuda et al. 2002; Nanda et al. 2002), similar to *Sry* in mammals. Interestingly, *dmrt1*, the ancestor of *dmrt1bY*, is one of the downstream effectors of SRY in the mammalian male pathway. The duplicated copy of *dmrt1* on the Y-chromosome has acquired an upstream position in the sex-determining cascade. Remarkably this evolutionary novelty, requiring a rewiring of the regulatory network, was brought about by co-optation of “ready-to-use” pre-existing *cis*-regulatory elements contributed by transposable elements (Herpin et al. 2010). With respect to their biochemical functions, both medaka *dmrt1* paralogs act as transcriptional regulators (Herpin et al. 2010). Dmrt1bY was shown to be responsible for male-speciﬁc primordial germ cell mitotic arrest in the developing gonad at the sex-determination stage (Herpin et al. 2007). In contrast, the autosomal *dmrt1a* medaka gene is essential for testis maintenance (Masuyama et al. 2012).

The medaka gonad is formed by the coordinated development of two different cell lineages: the germ cells and the somatic gonadal mesoderm surrounding the germ cells. Shortly before hatching, at the time of expression of *dmrt1bY* in the male gonad primordium, the germ cells in the female gonad actively proliferate and undergo meiosis, whereas this is not observed in male gonads (Kobayashi et al. 2004; Herpin et al. 2007). It is only 10 days later that the first somatic gonadal dimorphisms are apparent with the formation of the acinus (the seminiferous tubule precursor) and the follicles in gonads of male and female, respectively. Interestingly, ovarian cords within the germinal epithelia of medaka ovaries have been recently characterized (Nakamura et al. 2010). These cords composed of somatic *sox9b*-expressing cells and mitotic *nos2*-expressing oogonia continually give rise to germ cells and form a stem cell niche referred to as the germinal cradle (Nakamura et al. 2010). These cradles containing germline stem cells contribute to the production of fertile eggs during the life cycle of the adult ovary.

Like in other vertebrates studied so far, many components of the classical repertoire of mammalian sex-determining genes could be inventoried in medaka as well (Matsuda 2005; Siegfried 2010). To elucidate the gene regulatory network that controls specification and patterning of the gonads in the medaka fish, in this study we report the in vivo expression dynamics of classical mammalian markers within the forming and the adult gonads in medaka.

Because of their important roles in initiating male or female gonadal development in mammals, the respective implications of either Dmrt1 or Foxl2 transcription factors were examined. Further on, two of the major signaling pathways central for early gonadal induction and maintenance in mammals, namely the canonical Hedgehog and Wnt4/β-catenin signaling pathways (Wilhelm et al. 2007; Liu et al. 2010; Franco and Yao 2012), were investigated.

The Hedgehog (HH) signaling pathway plays an essential role in a wide variety of developmental processes (Ingham and McMahon 2001). Three HH proteins have been identified in mammals; SONIC (SHH), INDIAN (IHH), and DESERT (DHH) (Hooper and Scott 2005). *Dhh* and *Ihh* are co-expressed in the adult ovary where they stimulate proliferation and steroidogenesis of theca cells (Spicer et al. 2009). *Dhh* is also required for maintenance of the male germ line and spermatogenesis in mice (Bitgood et al. 1996; Clark et al. 2000). In mammals all three HH ligands signal by binding to one of two homologous transmembrane receptors, PATCHED homolog 1 and 2 (PTCH1 and 2). HH signaling is modulated by HH-induced transcription of the HH antagonistic interacting protein (HHIP) that binds to HH ligands and prevents their interaction with PTCH receptors (Chuang and McMahon 1999).

*R-spondin1* (*Rspo1*), a member of a small family of secreted growth factors, is a key female-determining factor. RSPO protein operates through the canonical WNT signaling pathway (Tomizuka et al. 2008) to activate the β-catenin pathway as well as via upregulation of *Follistatin* (*Fst*) through WNT4 (Yao et al. 2004). It is well established that mammalian R-SPO-1, WNT4, β-catenin, and FST are components of a single pathway that promotes ovarian development and suppresses the formation of testis cord (Chassot et al. 2008).

The winged helix/forkhead transcription factor FOXL2 is mainly expressed in the somatic cells of the female gonad (Crisponi et al. 2001). The major role of *Foxl2* during gonadal differentiation and maintenance has recently been shown via the mutual antagonistic relationship of *Foxl2* and *Dmrt1*. FOXL2 suppresses expression of *Dmrt1* and vice versa for maintaining female or male gonadal fate, respectively (Uhlenhaut et al. 2009; Matson et al. 2011). Additionally it has been reported that FOXL2 and WNT4 (Yao 2005; Ottolenghi et al. 2007; Garcia-Ortiz et al. 2009) cooperate in regulating FST expression during ovarian development. Interestingly in the ovary the expression profiles of *Foxl2* highly correlate with that of *Aromatase* (*Cyp19*), suggesting that *Foxl2* is involved in the regulation of estrogen synthesis via direct transcriptional upregulation of *Aromatase* (Pannetier et al. 2006). On the other hand, several other factors (e.g., testosterone, TGF-β1, TNF-α, and glucocorticoids) have been shown to direct the expression of the aromatase gene in Sertoli, Leydig, and germ cells of rat testis (see Bourguiba et al. 2003 for review).

Analyzing several fluorescent reporter lines established from a bacterial artificial chromosome recombination (BAC recombination) method resulting in optimal spatial resolution and high reliability of gene expression (Giraldo and Montoliu 2001; Nakamura et al. 2008b; Suster et al. 2011), we find major discrepancies between mammals and medaka, notably amongst spatial and temporal expression patterns of the canonical signaling pathways. Using in vivo whole tissues chromatin immunoprecipitation and in vitro gene regulation assays, we can reveal possible interactions between these pathways that emphasize the importance of the cellular context on modulating these regulations and call into question a strict conservation of regulatory and functional interactions of sexual development genes in vertebrates.

## Results

### *Patched-2* Expression and Hedgehog Pathway Regulation

To determine the role of the hedgehog signaling pathway in gonadal development of medaka, we recorded the temporal and spatial expression patterns of the key receptor, Ptch2.

**Fig. 1.**
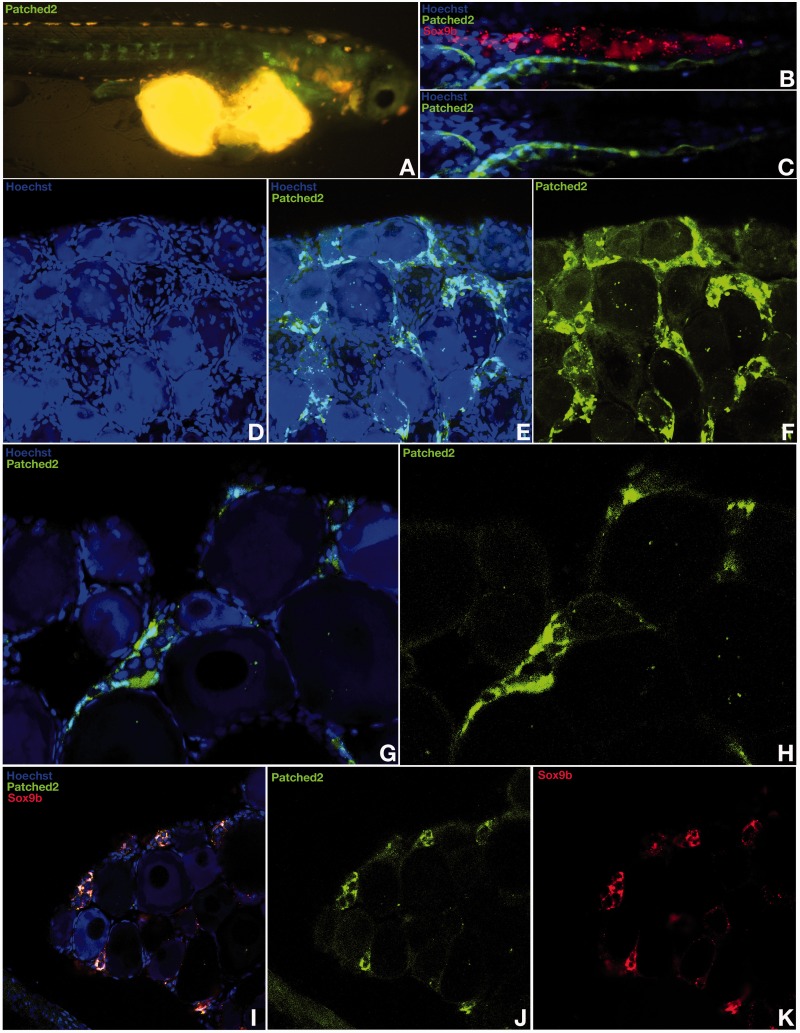
Spatial and temporal expression patterns of *patched-2* during medaka early gonad formation and in adult gonads. A *patched-2* BAC reporter transgenic medaka line expressing GFP was established to follow *patched-2* expression dynamics in vivo during gonad formation. Although expressed early on during somatogenesis (*A*), *patched-2* expression was never detected during the early phase of gonadogenesis at hatching stage (*B* and *C*). In the young ovary, *patched-2* expression is restricted to somatic cells of the ovarian cord (*D–H*) where it is co-expressed together with *sox9b* (*I–K*).

**Fig. 2.**
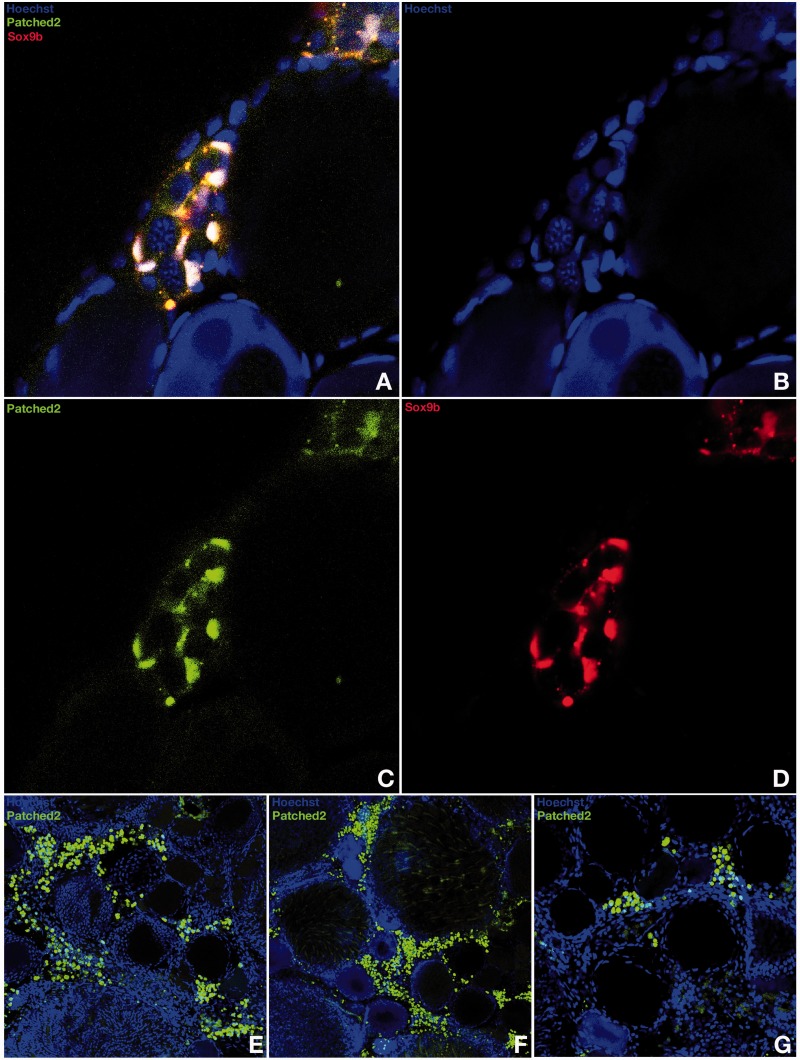
Specific *patched-2* expression in the germinal cradle of the ovary. *Patched-2* expression is specifically restricted to the somatic cells that express *sox9b* and surround the germ line stem cells of the germinal cradle within the ovarian cord but is absent in the germ line stem cells (*A–D*). In ovaries of fish of later reproductive phase, *patched-2* expression is only apparent in the interstitium (*E–G*).

**Fig. 3.**
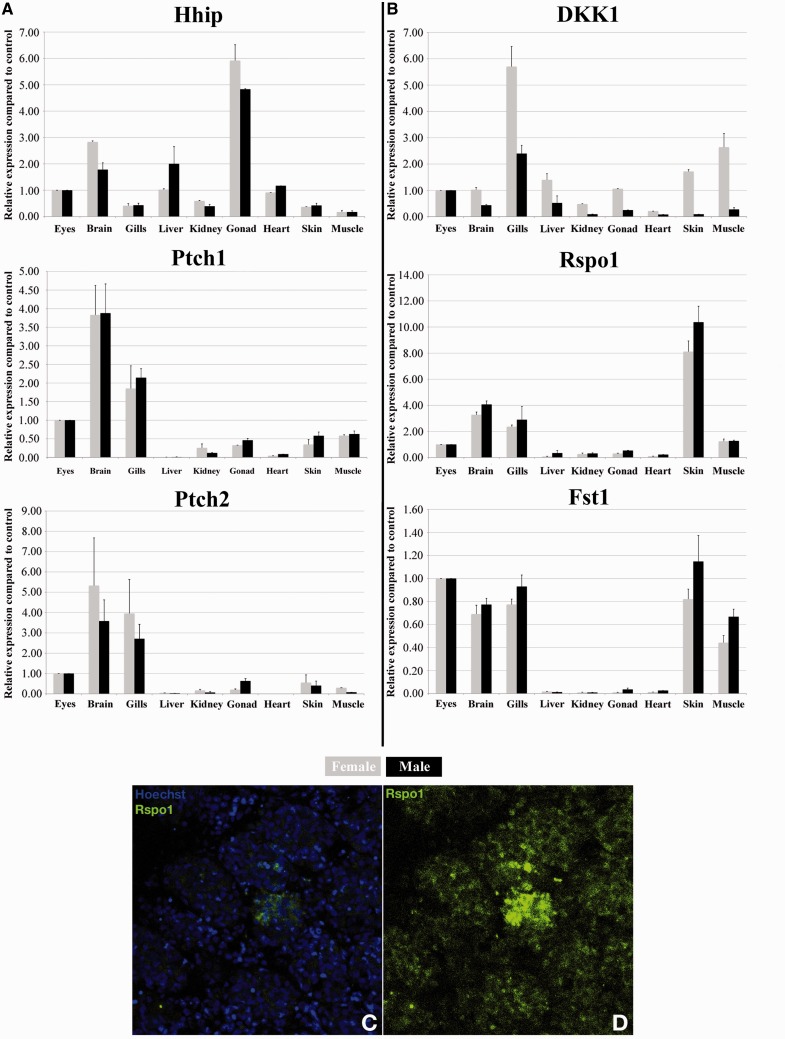
Real-time PCR expression patterns of Hedgehog and Wnt pathway components and in vivo reporter expression of *R-spondin1* in medaka adult testis. Expression patterns of different components of the Hedgehog (*A*) and Wnt (*B*) pathways in organs of adult male and female medaka determined from pooled (3–4 animals) total RNA extracts. In adult testes, background levels of *R-spondin1* expression are detected either by real-time PCR (*B*) or BAC reporter fluorescence (*C* and *D*) methods.

Although expressed quite early on during somatogenesis (data not shown), *patched-2* was surprisingly neither detected throughout the early phase of gonadogenesis when the undifferentiated gonad anlage grows up to hatching stage (fig. 1*A*) nor further on up to the stage when the dimorphic gonad develops 10 days after hatching (fig. 1*B* and *C*). The first specific gonadal *ptch-2* expression was detected in the young ovary (fig. 1*D*–*H*). Here expression is restricted to the somatic cells that express *sox9b* and surround the germline stem cells of the germinal cradle within the ovarian cord (Nakamura et al. 2010) (fig. 1*G* and *H*). Interestingly, *r-spo-1* is also co-expressed with *sox9b* in these cells (figs. 1*I*–*K* and 2*A*–*D* compared with fig. 8*A*–*F*). In ovaries of fish of later reproductive phase, when the cradle number has declined, *ptch-2* expression could be only noticed in the interstitium (fig. 2*E*–*G*). Unexpectedly and in contrast to *patched* expression in mammals, *ptch-2* expression was only detected at background levels throughout stages of testis development (data not shown). This inconspicuous role for the Hedgehog signaling in gonad development and maintenance was also apparent in real-time polymerase chain reaction (PCR) analysis. Although similarly expressed in different tissues, both *patched* receptor transcripts (1 and 2) are only expressed at background levels in adult gonads of both sexes (fig. 3*A*).

#### In vivo Dmrt1bY Binding to hhip Promoter Region

**Fig. 4.**
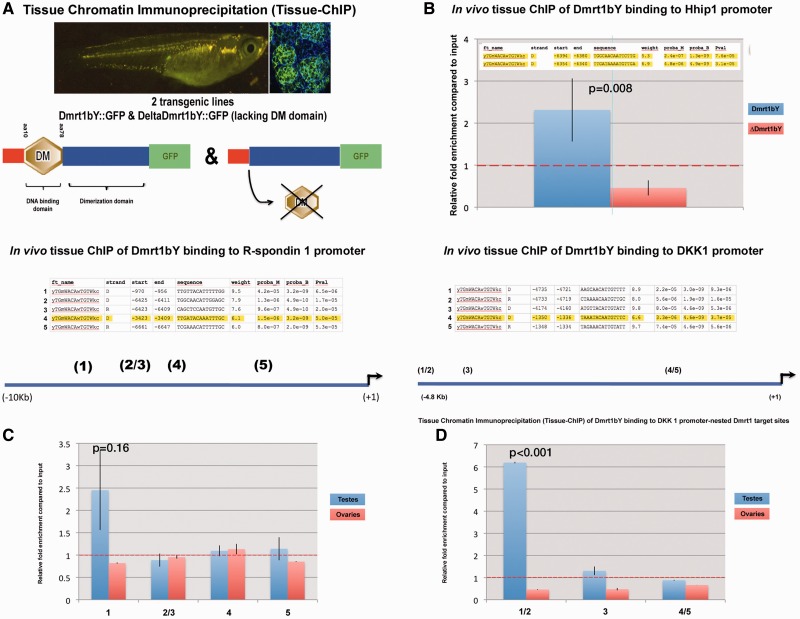
In vivo tissue chromatin immunoprecipitation (in vivo tissue-ChIP) analysis of Dmrt1bY targets. Chromatin immunoprecipitation using both Dmrt1bY::GFP and deltaDmrt1bY transgenic lines respectively expressing either Dmrt1bY or as control a truncated Dmrt1bY (delta DM form lacking the DNA-binding domain) fused to GFP revealed in vivo specific Dmrt1bY protein affinities to target sites nested within *hedgehog interacting protein 1* (*hhip1*), *r-spondin-1*, and *dkk-1* respective promoter regions. (*A*) Transgenic lines established for in vivo tissue-ChIP. (*B–D*) Specific enrichment of *hhip-1* (*B*), *r-spondin-1* (*C*), and *dkk-1* (*D*) promoter-nested Dmrt1 binding sites subsequent to Dmrt1bY immunoprecipitation.

Given the pivotal role of Dmrt1 transcription factors during medaka sex determination, *patched-2* and the antagonistic regulatory HH interacting protein (*hhip*) promoter regions were scanned for putative Dmrt1bY and Dmrt1a target sites. Although no Dmrt1-binding sites could be identified in the *patched-2* promoter region (10 kb upstream scanned), two sites were predicted with high fidelity in the *hhip* 5′ region (fig. 4*B*). To assess the in vivo relevance of the predicted Dmrt1 interaction, two stable transgenic lines expressing either the full-length Dmrt1bY protein (Dmrt1::GFP) or a truncated form lacking the DNA-binding domain (ΔDmrt1::GFP), both fused to GFP, were utilized (Herpin et al. 2010). These two lines were used for in vivo tissue chromatin immunoprecipitation (in vivo tissue ChIP) on testis tissue using GFP antibody for immunoprecipitation (fig. 4*A*). For the predicted two proximal Dmrt1-binding sites in the *hhip* promoter, a more than 2.3-fold enrichment after immunoprecipitation validated Dmrt1 binding (fig. 4*B*). Under the same conditions, no binding was detected in ovary (data not shown). This indicates that, in vivo, Dmrt1bY and/or Dmrt1a are potentially regulating the gonadal HH signaling through direct transcriptional regulation of the antagonist *hedgehog interacting protein*, *hhip*.

#### Dmrt1-Induced Hedgehog Pathway Regulation

**Fig. 5.**
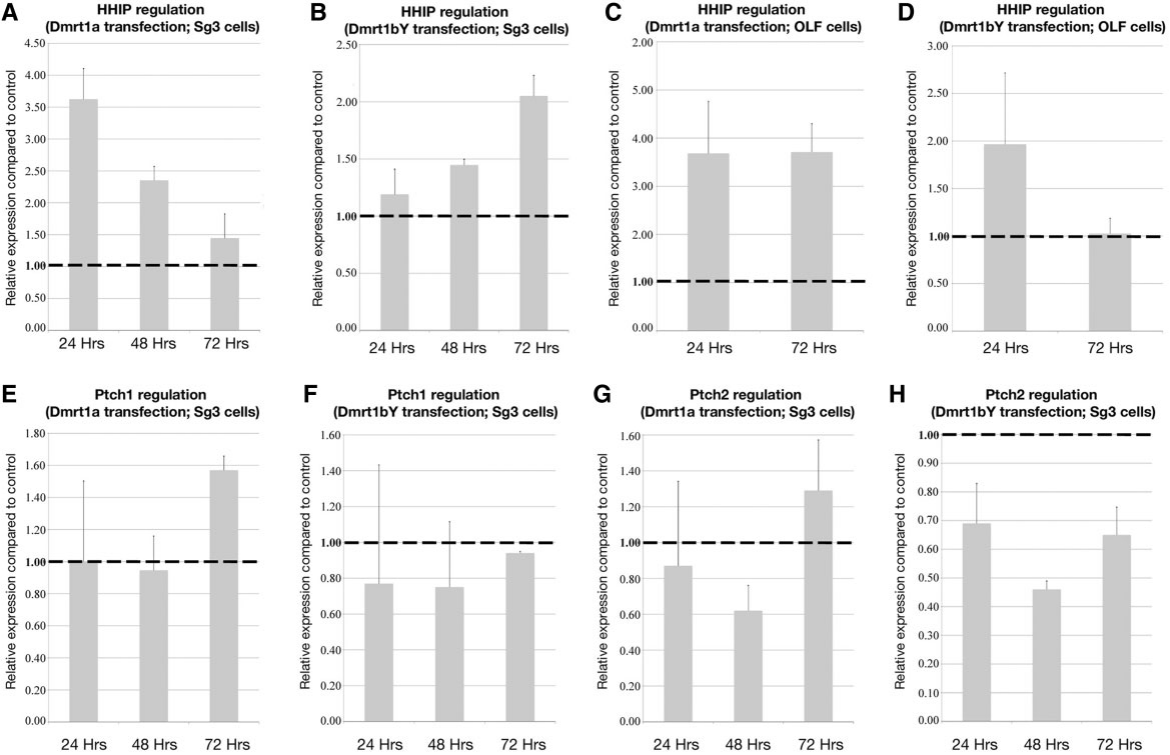
Hedgehog pathway transcriptional regulation after Dmrt1a/Dmrt1bY overexpression in different cell lines. *hhip* (*A–D*), *patched-1* (*E* and *F*), and *patched-2* (*G* and *H*) expression were monitored in different cell lines (SG3 and OLF) after Dmrt1a or Dmrt1bY overexpression. *Hhip* transcription is mainly upregulated by both Dmrt1s (*A–D*). *Patched-2* transcription is only clearly downregulated by Dmrt1bY (*G* and *H*) and *patched-1* expression levels remain unaffected (*E* and *F*).

Further functional characterization of Dmrt1-induced *hhip* transcriptional regulation was performed by overexpression of Dmrt1a or Dmrt1bY in spermatogonial (SG3) or fibroblast (OLF) cell lines of medaka. In both cell lines, *hhip* transcription was clearly induced (fig. 5*A*–*D*). Consistently, examination of the *hhip* expression pattern disclosed high and specific expression in testes (fig. 3*A*). Interestingly, at the receptor level (*patched 1* and *2*), although no direct interaction with Dmrt1bY could be demonstrated, transcriptional down regulation of *patched-2* was observed after Dmrt1bY overexpression in the spermatogonial cell line (fig. 5*E*–*H*). In line with the in vitro regulation data, *patched-1/2* expression in gonads of both sexes was not above background (fig. 3*A*). Due to the absence of the *dmrt1bY* gene in females, and since a background level of *dmrt1a* expression is detected in ovary (Hornung et al. 2007), the high expression of *hhip* and the suppression of *ptch-2* must be exclusively regulated by the autosomal *dmrt1a* ortholog in the ovary of medaka.

### *R-spondin 1* and *Follistatin* Expression and Wnt Pathway Regulation

Although ubiquitously expressed at early stages of development, neither medaka *r-spo-1* nor *fst* expressions could be detected in the presumptive gonadal mesoderm before the PGCs reach the undifferentiated gonadal primordium at stage 30 (fig. 7*A*–*C*; supplementary fig. S1, Supplementary Material online). Subsequently, in the dorsal region of the hindgut, a very furtive and time restricted pulse of *r-spo-1* and *fst* expression appears in the male gonadal primordium between stages 33 and 35 (fig. 7*D*–*F*). Interestingly, this very brief pulse of *r-spo-1*and *fst* expression occurs shortly before the rise of *dmrt1bY* expression at that stage (Kobayashi et al. 2004; Hornung et al. 2007). At hatching stage when sex determination occurs, no *r-spo-1*or *fst* expression could be detected in the gonadal primordia of males and females (fig. 7*G*–*I*). This lack of later gonadal expression is in line with similar findings in zebrafish (Zhang et al. 2011), turtle (Smith et al. 2008), chicken (Smith et al. 2008), and mice (Yao et al. 2004; Parma et al. 2006). Further on, while the ovary develops in juvenile females, *r-spo-1* expression is restricted to few somatic cells surrounding germline stem cells of the germinal cradle within the ovarian cord (fig. 8*A*–*F*). These somatic cells of the germinal cradle are the same that also co-express *sox9b* and *patched-2* (fig. 2*A*–*D* compared with fig. 8*A*–*F*). Similar to zebrafish (Zhang et al. 2011) and mouse (Smith et al. 2008), expression of medaka *r-spo-1* was also detected in granulosa cells around young oocytes (fig. 8*G*–*L* and table 1). Granulosa expression is then progressively lost while the oocytes are growing. In male gonads, only a low expression of *r-spo-1* is detected (fig. 3*B*–*D*). Medaka *fst* could not be detected in the ovary at this stage. In contrast to germ cell expression of *r-spo-1* in chicken (Smith et al. 2008), zebrafish (Zhang et al. 2011) and mouse (Smith et al. 2008) ovaries or zebrafish testis (Zhang et al. 2011) or *fst* in rat testis (Meinhardt et al. 1998), neither *r-spo-1* nor *fst* could be detected in gonadal germ cells or spermatogonia in medaka (fig. 8 and table 1). In older ovaries, *r-spo-1* and *fst* are also expressed in the interstitium as well as in the ovarian epithelium (fig. 8*M*, *N*, and *Q*–*S*). Of note, sparse clusters of *fst* expressing interstitial somatic cells were detected in testes (fig. 8*O* and *P*).

**Table 1.** Comparative Analysis of Gonadal Expression of R-spondin1, Follistatin, Patched2, and Foxl2 in Vertebrates.

Genes		Gonadal Expression Patterns (Adult Ovaries/Testes)		
		Medaka	Mouse	Other Vertebrates
***R-spondin 1***	O	(A) Somatic cells surrounding the germ cells and germline stem cells of the ovarian cord. Granulosa cells of the young oocytes. (E) Not expressed during early gonadal development.	(E) Predominantly in the somatic cells of the developing ovary (Smith et al. 2008). (E) Germ cells (during meiosis at low levels) (Smith et al. 2008).	Chicken: (E) outer cortical zone of the developing ovary and germ cells during meiosis (Smith et al. 2008). *Danio*: (A) granulosa and theca cells. Premature germ cells, oogonia, primary oocytes (Zhang et al. 2011).
	T	N.D.	N.D. (Smith et al. 2008).	Chicken: (E) not detected (Smith et al. 2008). *Danio*: (A) Leydig cells, spermatogonia, and spermatocytes (Zhang et al. 2011).

***Follistatin***	O	(A) Ovarian eptithelium and interstitium of old ovaries.	(E) Somatic cells of the embryonic ovary (Menke and Page 2002; Yao et al. 2004), (A) co-localization with Foxl2 (Kashimada et al. 2011).	Sheep: (A) granulosa cells of the growing follicles II and III (Tisdall et al. 1994).
	T	N.D.	(E) Not detected in embryonic testes (Menke and Page 2002).	Rat: (A) Sertoli and endothelial cells, germ cells, spermatogonia, spermatocytes, and round spermatids (Meinhardt et al. 1998).

***Patched 2***	O	(E) Not expressed during early gonadal development. (A) Somatic cells surrounding the germ cells and germline stem cells of the ovarian cord (co-expression with r-spondin1). Additional expression in the interstitium of the old ovaries.	(A) Highly expressed (testis-specific splice variants) (Szczepny et al. 2006).	Tammar wallaby: (E) expressed throughout the development of the embryonic ovary. (A) Abundant in granulosa, cumulus, and theca cells of the adult ovary. Very weak in germ cells (O'Hara et al. 2011).
	T	N.D.	(A) Lowly expressed (Spicer et al. 2009).	Tammar: (E) Leydig cells in the interstitium of the developing testes. (A) Restricted to Sertoli cells of the adult testes (O'Hara et al. 2011).

***FoxL 2***	O	(A) Ovarian germline stem cells, initial stage of post meiotic oocytes. Sub-population of theca and granulosa cells together with aromatase (Cyp19a1) expression.	(E) From 12.5 dpc in mesenchymal pre-granulosa cells and (A) later in granulosa cells (Schmidt et al. 2004). (A) Small and medium size follicles (Pisarska et al. 2004).	Chicken: (A) medullar part of the ovary, maturing and ovulated oocytes. Granulosa cells, weak in theca cells layer (Govoroun et al. 2004).
	T	N.D.	N.D.	

Note.—(E) embryonic expression; (A) adult expression.

#### In vivo Dmrt1bY Binding to the r-spo-1 and dkk1 Promoter Regions and Regulation of the r-spo-1 Pathway

**Fig. 6.**
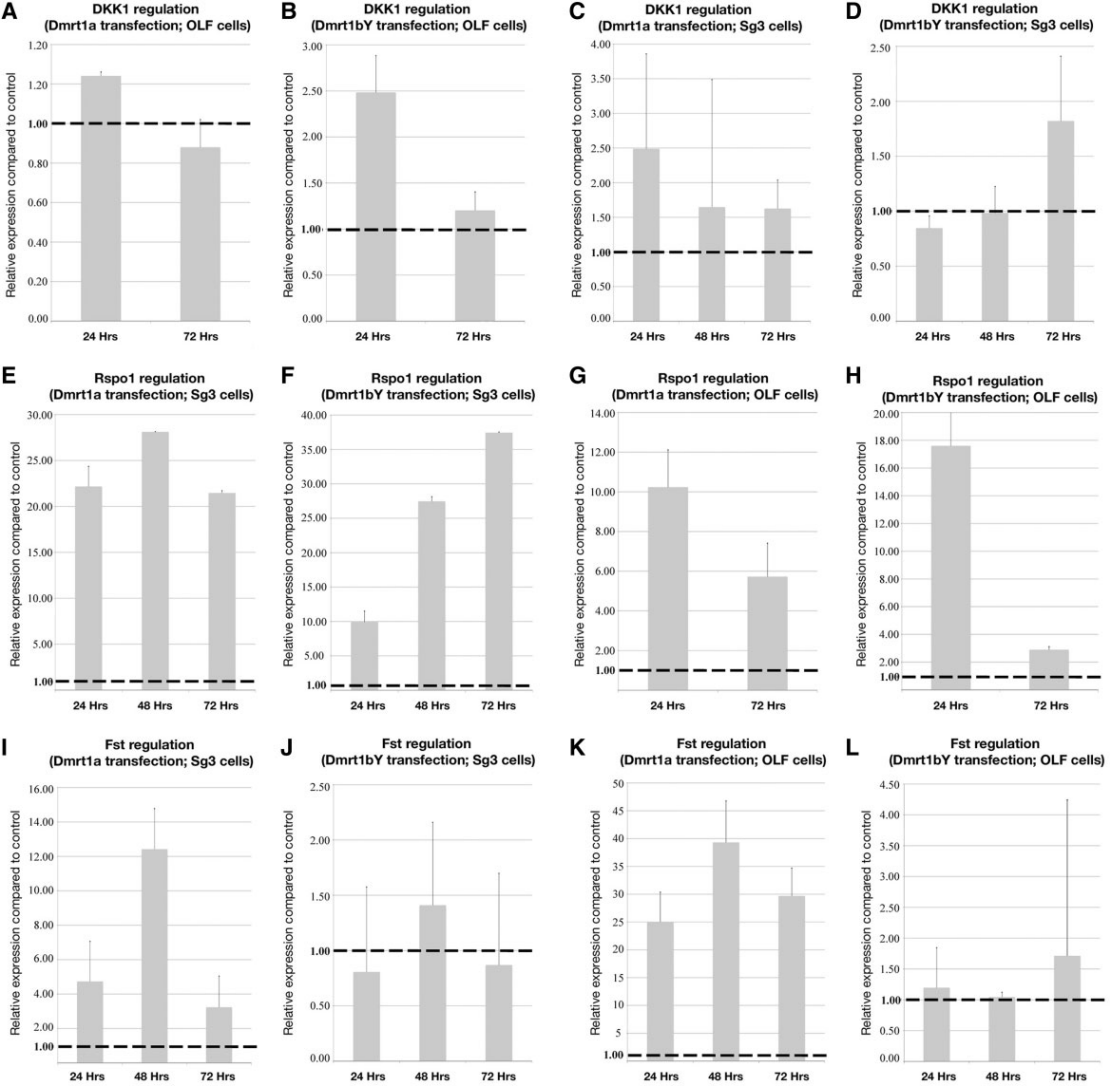
Canonical *Wnt* pathway (*dkk-1*, *r-spondin-1*, and *fst*) transcriptional regulation after Dmrt1a/Dmrt1bY overexpression in different cell lines. *dkk-1* (*A–D*), *r-spondin-1* (*E–H*), and *fst* (*I–L*) expression was monitored in different cell lines (SG3 and OLF) following Dmrt1a or Dmrt1bY overexpression. Transcription of dkk-1 and r-spondin-1 is upregulated by both Dmrt1s (*A–H*). Differential regulation of *fst* is observed. While no regulation was observed after Dmrt1bY overexpression, *fst* transcription is clearly upregulated in both cell lines overexpressing Dmrt1a (*I–L*).

We next analyzed the capacity of Dmrt1 to transcriptionally regulate positive (*r-spo-1* and *fst*) and negative (*dkk1*) effectors of the Wnt/Rspo1/Fst pathway. Hence, the ability of Dmrt1 to directly bind in vivo to the *fst*, *r-spo-1*, and *dkk1* promoters was investigated. Out of several putative Dmrt1-binding sites within the *r-spo-1* and *dkk1* promoter regions, in vivo ChIP revealed direct Dmrt1bY interaction with both promoters (fig. 4*C* and *D*). Of note, robust binding of Dmrt1bY to *dkk1* promoter region was seen in testes, but similar interactions did not occur in ovaries (fig. 4*D*). Although not highly significant, an analogous trend is observed for Dmrt1bY binding to *r-spo-1* promoter (fig. 4*C*). Furthermore cell transfection experiments overexpressing either Dmrt1a or Dmrt1bY showed a Dmrt1bY-specific slight transcriptional upregulation of *dkk1* (fig. 6*A*–*D*). Interestingly, although *fst* does not seem to be under Dmrt1bY regulation (fig. 6*J* and *L*), Dmrt1a does upregulate *fst* transcription (fig. 6*I* and *K*). Astonishingly and in contrast to our in vivo expression data, Dmrt1-induced transcriptional upregulation was observed for *r-spondin-1* (fig. 6*E*–*H*). Real-time PCR quantification nevertheless revealed *r-spo-1*and *fst* to be only expressed at background levels in gonads (fig. 3*B*), surprisingly also including adult testis (fig. 3*C* and *D*).

**Fig. 7.**
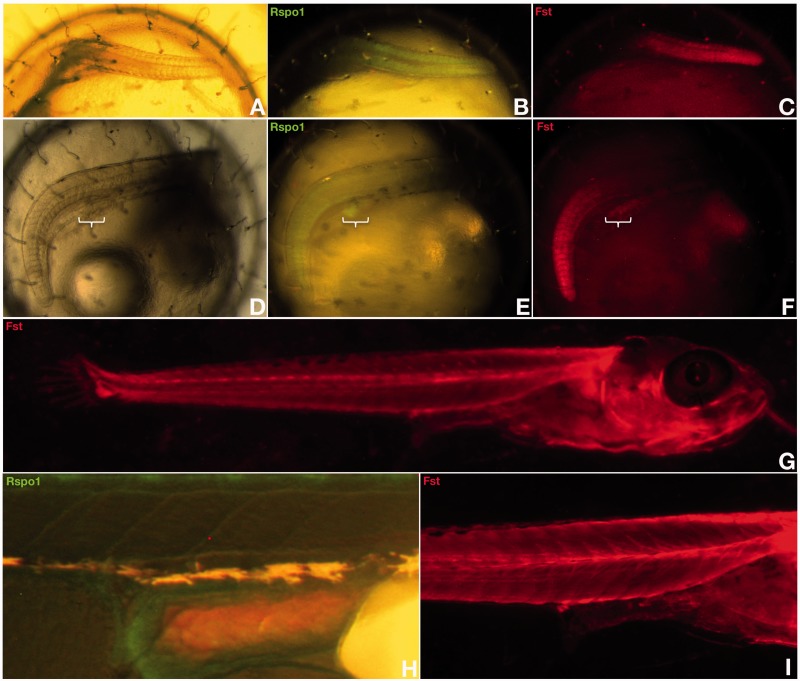
*R-spondin-1* and *follistatin* spatial and temporal expression patterns during medaka gonad primordium formation. Two BAC reporter fish transgenic lines expressing either GFP or mCherry were established to follow r-spo-1 and fst expression dynamics during gonad formation in vivo. Neither *r-spo-1* nor *fst* expression was detected in the presumptive gonadal mesoderm at early stages of development (*A–C*). Between stages 33 and 35 in the dorsal region of the hindgut *r-spo-1* and *fst* are co-expressed (*D–F*). At hatching stage, although ubiquitously expressed, neither *r-spo-1* nor *fst* were detected in the gonadal primordium (*G–I*).

### Foxl2 Expression in the Adult Gonad

To investigate the role of Foxl2 in ovarian differentiation, we analyzed Foxl2 protein distribution in the medaka ovary (figs. 9 and 10). During the transition process of germ line stem cells to oocytes within the germinal cradle, medaka Foxl2 expression starts within the germ line stem cells and continues during meiosis until early oogenesis (fig. 9*A*–*L*). On the contrary, no Foxl2 protein could be detected in the interwoven threadlike ovarian cords of *sox9b*-expressing cells where the supporting follicular cells reside (fig. 9*A*–*L*). During the following steps of oogenesis, the accompanying cells of the supporting layer progressively loose *sox9b* expression while Foxl2 expression rises (fig. 9*A*–*L*). Consistent with mRNA localization (Nakamoto et al. 2006), Foxl2 protein in the medaka ovary was localized within the follicular cells of the previtellogenic and vitellogenic follicles and then gradually lost while maturation proceeds (fig. 10*A*). Particularly, Foxl2 is present in the nuclei of all granulosa cells (fig. 10*B*–*D*). Interestingly, while Foxl2 has been reported to be a strong inducer of the steroidogenic activity of granulosa cells via upregulation of the aromatase gene (the ovarian-type Cyp19a1) (Hudson et al. 2005; Wang et al. 2007; Guiguen et al. 2010), unexpectedly, and in contrast to mammals, a minority of theca cells do also express Foxl2 in medaka (fig. 10*E*–*G*). Our results reveal two subpopulations of *cyp19a1*-positive theca cells, which are either Foxl2 positive or do not express the transcription factor (fig. 10*H*–*K*).

**Fig. 8.**
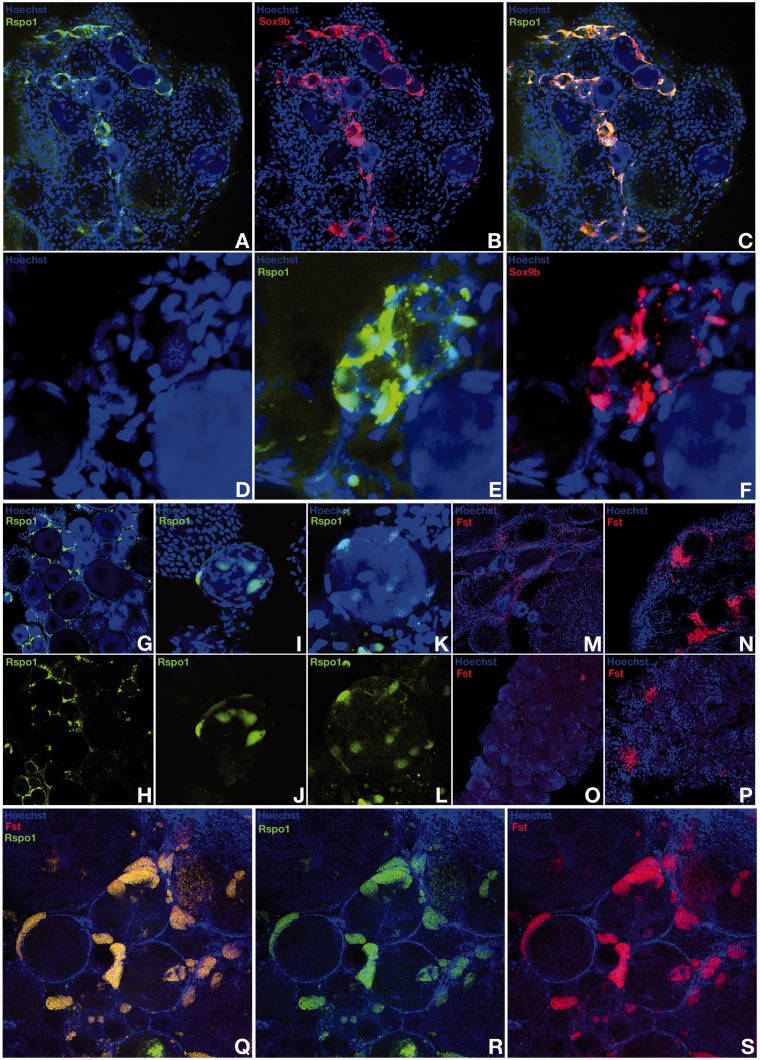
*R-spondin-1* and *follistatin* expression in the adults gonads. In the ovary of juvenile females, *r-spondin* expression is restricted to somatic cells surrounding the germ line stem cells of the germinal cradle within the ovarian cord (*A–F*). Co-expression with *sox9b* (*A* to *F*) and *patched-2* (see fig. 2*A–D*) is observed. *R-spondin-1* expression in granulosa cells is also detected around young oocytes (*G–L*). In the ovary, *follistatin* expression was only detected in the interstitium (*M* and *N*) and in the ovarian epithelium together with *r-spondin-1* (*Q–S*). Sparse clusters of *follistatin* expressing interstitial somatic cells are also detected in adult testes (*O* and *P*).

**Fig. 9.**
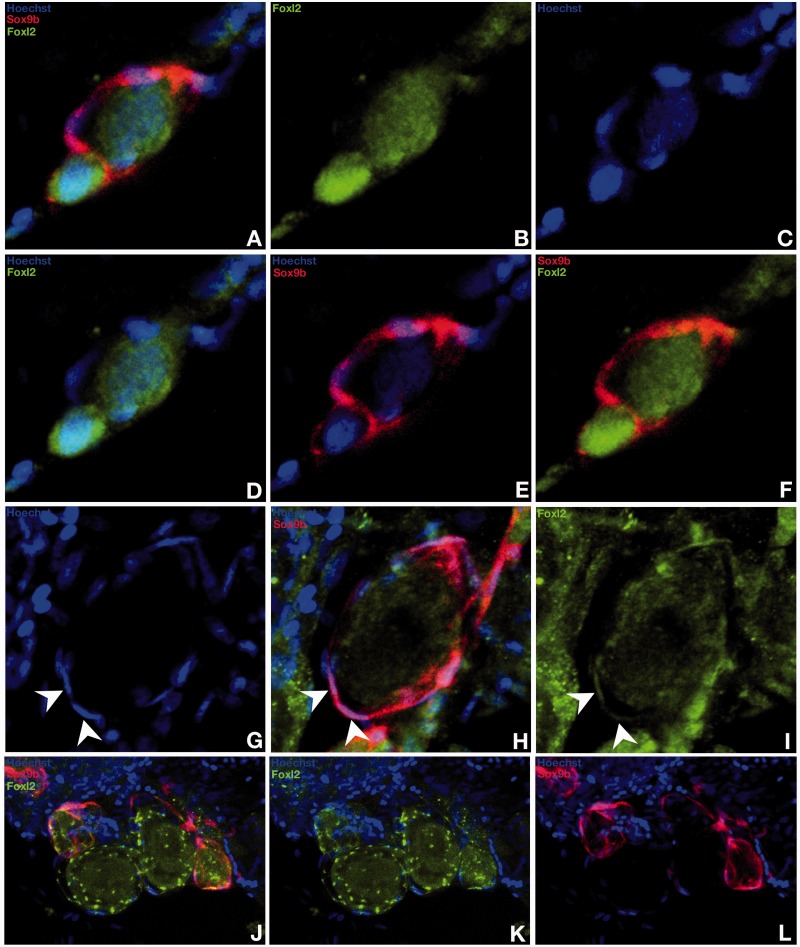
*Foxl2* protein localization in the ovarian cradle. During development from germ line stem cells to oocytes within the germinal cradle, foxl2 localization is first detected in the germ line stem cells and remains during meiosis until early oogenesis (*A–L*). Concomitantly, the accompanying somatic cells of the supporting layer progressively loose *sox9b* expression while *foxl2* expression rises (*A–L*).

**Fig. 10.**
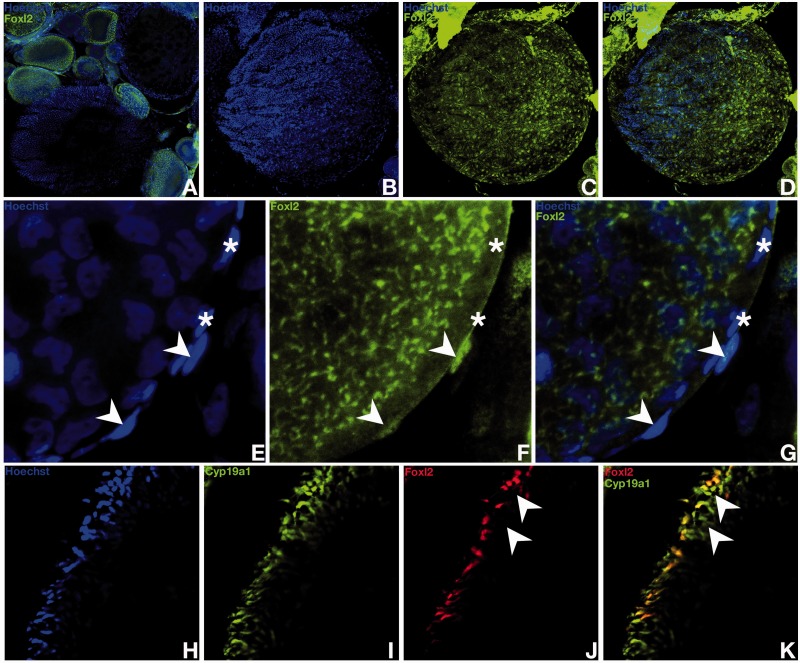
Protein expression and localization of *foxl2* in the ovary. *foxl2* immunostaining is present in the nuclei of the follicular cells of the previtellogenic and vitellogenic follicles (*A*). In vitellogenic follicles, *foxl2* protein is detected in all granulosa cells (*B–D*). Nuclear localization of *foxl2* in few theca cells (*E–G*). *foxl2* expression occurs only in a sub-population of theca cells (arrow heads vs. asterisk in *E–G*) as shown by comparison with the thecal layer marker aromatase *cyp19a1* (*H–K*).

## Discussion

Comparative studies on sex determination cascades of different organisms revealed that the genetic control of male or female gonad development displays between different groups of organisms a remarkable diversity of “master sex-determining genes” at the top of the genetic hierarchies, whereas downstream components surprisingly were found more widespread and are evolutionarily more conserved. Without much further studies, these observations led to the reasoning that conservation of sequence equalizes to conservation of function. While vertebrates have at least a common set of transcriptional regulators, including DMRT1 and FOXL2, as well as some signaling molecules and pathways such as the Hedgehog and R-spo-1/Wnt4 pathways, their molecular interplay and epistatic relationships are nevertheless far from being understood. The purpose of our work was to examine this molecular interplay in fish.

### Absence of *R-spo-1*, *fst*, and *ptch-2* in Medaka Germ Cells

In the mouse it was proposed that activation of the R-spo-1/Wnt/Fst signaling pathway in both somatic and germ cells, besides triggering meiosis in fetal germ cells, is required for ovarian differentiation and maintenance of ovarian cell identity (Chassot et al. 2008). Indeed inherent to their inductive epigenetic mode of specification, the germline sex is likely to be determined early on by *Sry* acting in the somatic cells (McLaren and Southee 1997; Sekido and Lovell-Badge 2008; Bowles et al. 2010) in a noncell-autonomous manner (see De Felici 2009 for review). *Sry* controls whether bipotential precursor cells differentiate into testicular Sertoli cells or ovarian granulosa cells (Koopman et al. 1991). This pivotal decision in a single gonadal cell type ultimately controls sexual differentiation throughout the body. Sex determination can be viewed as a battle for primacy in the fetal gonad between a male regulatory gene network in which *Sry* activates *Sox9* and a female network involving WNT/β-catenin signaling (Uhlenhaut et al. 2009; Herpin and Schartl 2011; Matson et al. 2011).

In contrast, the sexual plasticity of medaka germ cells seems to be retained much longer than in mammals as illustrated by XX/XY transplantation chimeras. Although XY somatic cells differentiate into male cells according to their sex chromosome composition, in this environment XX germ cells differentiate into male cells regardless of their sex chromosome composition (Shinomiya et al. 2002). Hence, while unlike their mammalian counterparts none of the different medaka marker genes analyzed (*r-spo-1*, *fst* or *ptch-2*) were detected in germ cells at any time of gonadal development, this major inconsistency between mammals and fish certainly reflects intrinsically divergent modes of germ cell commitment and interaction between germ and somatic cells possibly accounting for a higher sexual plasticity of germ cells in fish.

### Expression of the *Rspo1*/*Fst* and Hedgehog Pathways

#### Role of the Rspo1/Wnt/Fst Pathway during Gonad Development and Maintenance

In line with observations made in zebrafish (Zhang et al. 2011), turtle (Smith et al. 2008), chicken (Smith et al. 2008), and mice (Yao et al. 2004; Parma et al. 2006), the absence in medaka of *r-spo-1* and *fst* dimorphic expression during sex determination stages does not support a role during gonad induction. Of particular interest and unlike in mammals, the lack of medaka *ptch-2* expression during the same period additionally rules out any involvement of the Hedgehog pathway for gonad induction. Further on, while the ovary develops in the juvenile female, the strict co-expression of *r-spo-1* and *ptch-2*, together with *sox9b* in the somatic cells of the germinal cradles, likely indicates a role in differentiating and specifying the somatic supporting lineage of the ovary. Of note the early decoupling of *r-spo-1 and fst* expression patterns, although expected to be involved in the same signaling pathway in mammals (Yao et al. 2004), de facto invalidates such an interplay in the medaka ovarian cradle.

Our results support the view that *r-spo-1* has a globally conserved female-specific expression profile in vertebrate gonads despite some slight but intriguing divergences. In all analyzed vertebrate species it is apparent that *r-spo-1* expression goes along with the process of somatic cell organization within the young ovary (table 1). In mouse, chicken, and medaka, *r-spo-1* expression at that time, predominantly or exclusively in the somatic cells, suggests its implication in a conserved pathway leading to folliculogenesis. On the other hand, the absence of medaka *r-spo-1* expression in gonad embedded germ cells, unlike in zebrafish, mice, chicken, and turtle, reveals that a role for germ cell development might not be accordingly conserved in the adult gonad of medaka.

In mammals, R-spo-1 engages the effector pathway of Wnt signaling and β-catenin and thereby activates Fst expression (Carmon et al. 2011). Hence, also medaka *fst* spatial and temporal expression pattern was expected to overlap with *r-spo-1*. While medaka *r-spo-1* and *fst* indeed display similar expression in adult tissues (fig. 3*B*), medaka *fst* was, however, absent during the early and late stages of ovarian induction and development. *Fst* expression was perceptible only in the interstitium and epithelium of old ovaries together with *r-spo-1* (fig. 8*M*, *N*, and *Q*–*S*). Unexpectedly, *fst* was also detected in testes (fig. 8*O* and *P*). This expression pattern indicates that in medaka and unlike in mammals, Fst is probably neither acting throughout early female gonad patterning nor during maintenance of cell identity in the adult ovary. Instead medaka *fst* expression appears to be more a marker of the aging ovary, probably acting during follicular atresia. Of interest, while exclusive ovarian expression is generally described for *R-spo-1* and *Fst* in vertebrates, such strict female dimorphism was not observed for *r-spo-1* and *fst* in zebrafish, rat, and medaka (table 1 and fig. 3).

#### Role of the Hedgehog Pathway during Gonad Development and Maintenance

The gonadal expression pattern of two components of the Hedgehog pathway in medaka is peculiar and different from what has so far been reported for other vertebrates including mammals (table 1). Unlike in mammals it appears that medaka gonadal HH signaling through the *patched-2* receptor is not involved in inducing and specifying the gonad primordia. It would rather act late exclusively in the process of somatic cell differentiation in the ovarian cradle. In strict contrast to its mammalian counterparts, the quasi-absence of medaka *patched-2* expression during testis formation in larvae and for testis cell identity maintenance in the adult rules out any functions during these processes (table 1). Certainly, the low testicular expression of *patched-2* together with Dmrt1a/1bY-induced transcriptional upregulation of the HH antagonist *hhip* indicates a general function of Dmrt1 in actively downregulating the Hedgehog pathway in medaka testes. Taken together, we can conclude that although apparently downregulated at the transcriptional level, a background expression of *patched-2* remains. This phenomenon is known as illegitimate transcription (Chelly et al. 1989; McLeod and Cooke 1989). We speculate that the high expression of the *hhip* hedgehog pathway antagonist in gonads (about 5 to 10 times higher than patched1/2 expression [fig. 3]) likely prevents any hedgehog activation resulting from leaky background expression of patched receptors. Interestingly, several putative Sox9 binding sites are present in the patched-2 promoter region and might explain the strict *sox9b*/*patched-2* co-expression observed in the supporting cells of the ovarian cradle.

### A Newly Identified Subpopulation of Theca Cells Expressing *Aromatase* but Not Foxl2

Examination of Foxl2 protein distribution in the medaka ovary allowed us to define a new subpopulation of theca cells expressing Foxl2. Also expressing *cyp19a1* (aromatase) these cells are then suspected of having a steroidogenic activity. In contrast to mammals where ovarian-type *aromatase* is only produced by granulosa cells, the biological significance of such cells expressing *cyp19a1* and Foxl2 within the thecal layer remains unclear. Of note, in ovaries of *cyp19a1/p450c17I* double transgenic medaka reporter lines, two subpopulations of theca cells were previously identified, being either *cyp19a1* or *p450c17I* positive in a mutually exclusive manner (Nakamura et al. 2009). The *cyp19a1* expressing subpopulation of theca cells was already considered as the precursors of the theca lineage (Nakamura et al. 2009).

**Fig. 11.**
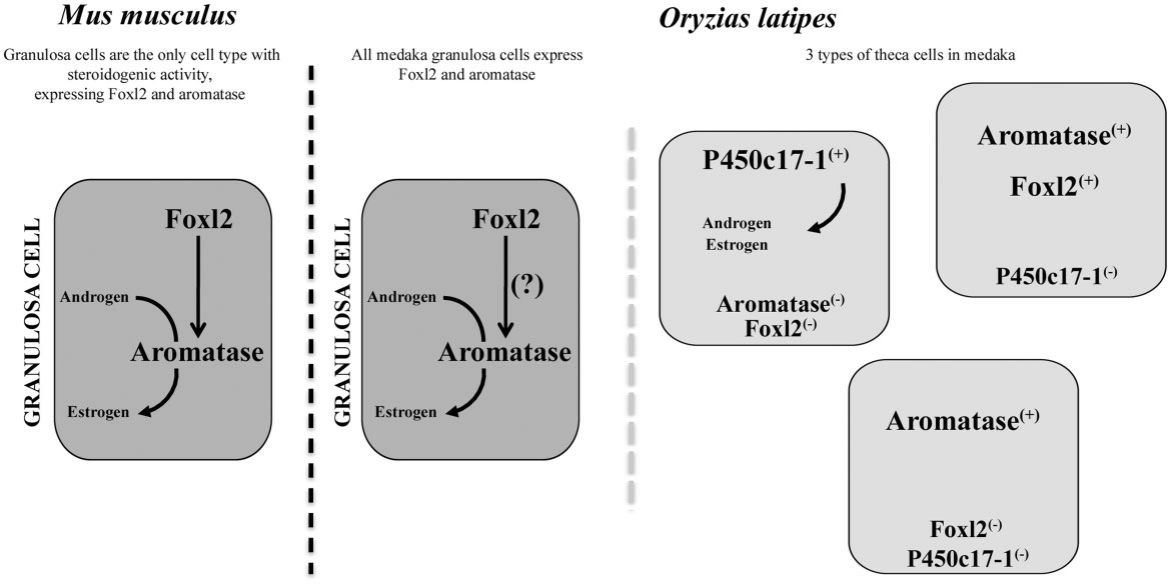
Schematic representation of granulosa and theca cell populations in mouse and medaka. In mammals, granulosa cells are the only cell type with steroidogenic activity, expressing both *foxl2* and *aromatase*. *Aromatase* expression is directly induced by *foxl2*. In medaka, like in mammals, granulosa cells express both Foxl2 and *aromatase*. Examination of Foxl2 protein distribution in the medaka ovary revealed a new subpopulation of theca cells expressing Foxl2.

The strict co-expression of *Foxl2* and *Aromatase* (*Cyp19*) in the mammalian ovary led to the further demonstration that *Foxl2* is involved in the regulation of estrogen synthesis via direct transcriptional upregulation of ovarian-type *Aromatase* (see Pannetier et al. 2006 for review). Surprisingly in medaka we found, within the thecal layer, aromatase-only positive theca cells that remained Foxl2-negative. In that perspective it is interesting to note that birds also have multiple populations of theca cells some of which are also steroidogenic (Nitta et al. 1991). In contrast to the main consensus, the discordance of spatial expression patterns of Foxl2 and ovarian-type *aromatase* (*cyp19a1*) calls into question an exclusive transcriptional regulation of *cyp19a1* by Foxl2 in the ovary of medaka (fig. 11). Although we cannot exclude that aromatase-only positive cells have not been previously also positives for Foxl2, implying the requirement of Foxl2 for the induction of the aromatase expression, our results indicate that foxl2 is nevertheless not required for the maintenance of the aromatase expression.

### Variable Molecular Interplay among the Repertoire of Gonadal Markers during Medaka Gonad Formation and Maintenance

**Fig. 12.**
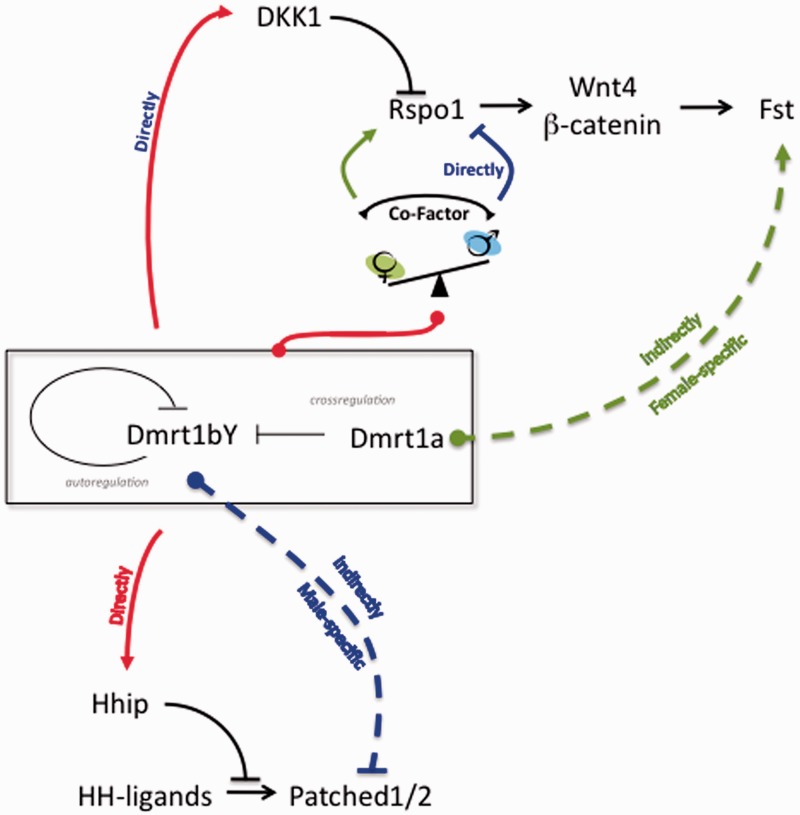
The *Dmrt1bY/Dmrt1a* gene regulatory networks during gonadal formation in medaka. Interaction scheme of the possible Dmrt1bY/Dmrt1a-triggered regulations of the Hedgehog and Wnt4 pathways during gonadal formation in medaka. Solid red arrows indicate for both Dmrt1bY and Dmrt1a positive regulation while dashed lines indicate sex-specific regulations. The green dashed line indicates a Dmrt1a, female-specific, indirect positive regulation favoring the expression of *follistatin*, while the blue dashed line reports a Dmrt1bY, male-specific, indirect repression of *patched-2* transcription. For the Dmrt1bY/Dmrt1a-triggered transcriptional regulation of *R-spondin1*, depending on the cellular contexts, the involvement of a sex-specific co-factor is proposed.

We could show a direct regulation of the Hedgehog and R-spo-1 pathways by Dmrt1bY (fig. 12). It is thus becoming apparent that despite its tangible requirement for mammalian testis formation and later on in regulating Leydig and myoid cell function (Clark et al. 2000; Pierucci-Alves et al. 2001; Canto et al. 2004), the Hedgehog pathway might not only be dispensable during medaka male gonadogenesis and maintenance but even needs to be suppressed as it is actively repressed by Dmrt1 genes. This would also explain the specific lack of *ptch-2* expression in medaka testes shown to be indirectly downregulated by Dmrt1bY (see fig. 12 for summary). In contrast the female-specific, Dmrt1a-triggerd upregulation of *follistatin* transcription might nevertheless point out the importance of the R-spo-1 pathway during female gonad differentiation although the upstream components of this same pathway are tightly regulated by the Dmrt1 co-orthologs (fig. 12).

Importantly, showing that under certain conditions Dmrt1 paralogs are able to strongly upregulate the female-specific r-spondin-1 gene expression, we could also demonstrate that Dmrt1bY/Dmrt1a-triggered regulations are highly dependent of the cellular context and might suggest requirement of co-factors (fig. 12). These findings are reminiscent of observations showing Stra8 transcription to be directly repressed by DMRT1 in mouse testes while activated in the fetal ovary (Krentz et al. 2011).

Interestingly, the canonical Wnt/ β-catenin (R-spo-1 and Fst) pathway strongly antagonizes FGF9, a robust component of the male sex-determining cascade in mammals (Kim et al. 2006; Matson et al. 2011). However, FGF9 is absent in the fish lineage and no indication of a redundant action of related FGF has been obtained, questioning the importance of FGF signaling in fish sex determination (Forconi et al. 2013). Into that direction the importance of the FGF9 signaling in vertebrates is actually also challenged by the finding that in chicken embryos *Fgf9* does not show any sexually dimorphic expression pattern during gonadal differentiation (Cutting et al. 2013). Similarly, although phylogenetically preserved, the Sox9 gene, a direct target of Sry in mammals, has been shown to be functionally dispensable for medaka testis determination (Nakamura et al. 2008a, 2012). In this context our data might reflect a profound reorganization of that part of the fish gonadal regulatory network compared with mammals. While some components such ase DMRT1, SOX9, FOXL2 and pathways such as Hedgehog or R-spo1/Wnt/Fst of the gonadal gene regulatory network are conserved on the DNA sequence level across phyla, their functions, regulation, and interplays might be considerably different.

## Materials and Methods

### BAC Recombination

Bacterial artificial chromosome clones encompassing medaka *patched-2* (ola1-199K19), *follistatin* (ola1-124N21), or *r-spondin-1* (ola1-158A23) genomic regions were obtained from NRBP Medaka (http://www.shigen.nig.ac.jp/medaka/, last accessed August 12, 2013). A BAC transgenic method using homologous recombination was employed to generate the reporter constructs as previously described (Nakamura et al. 2008b; Herpin et al. 2010). The following primers were used to amplify *eGFP/mCherry* fragments for homologous recombination into the different BAC clones: BAC-Ptchd2-GFP-Fw: GCTGAACTCGCACCGATTCTGCGTCGCCTCCTGTTACCCGTCTTTGGACTATGGATATCATTTCTGTCGCCTTAAAG, BAC-Ptchd2-GFP-Rv: CGCAAGCGGCTGGGAGCGCGTATAACTCGGGGGTAAATCTCCAAAGACGCCAGAACAAACGACCCAACACCGTGCG; BAC-Fst-Cherry-Fw: CTTTTGCGCTGCTTGTGTCAAATACGTGGCTCACTTTGCCTCTCCATCATGCTTGGGCCACCGGTCGCCACCATGGT, BAC-Fst-Cherry-Rv: CTTACCTTGAACCTTCTGATGTTCCATGAGGTGACAAAGCCACATGAAGAAGAGAGTCGACCAGTTGGTGATTTTG; BAC-Rspo1-GFP-Fw: GATCCATCTGGTTGCAGGGGGGGACCTTGCACAGCCTGGAAGGCAGCAGGGACTCCACCGGTCGCCACCATGGTG, BAC-Rspo1-GFP-Rv: CTTCTCGCCTTGGAGAGTTTGACAACATCGCTGTGACCCATGGAGCTGAGAATGAGTCGACCAGTTGGTATTTTG. After homologous recombination, the generated fragments were inserted into the BAC clones in frame downstream of the translation initiation site of the targeted genes.

### Generation of BAC Transgenic Medaka Lines and Imaging Analyses

The Carbio (WLC# 2674) strain of medaka (*Oryzias latipes*) was used for establishment of the transgenic lines. Microinjection of DNA was performed as described previously (Herpin et al. 2009) using BAC clone DNA at a concentration of 50–100 ng/mL. Adult G0 fish were then screened for fluorescence, and positive individuals were raised to adulthood. Siblings from positive G0 fish were mated to each other and the offspring were again sorted for fluorescence. *Sox9b* and *cyp19a1* (*aromatase*) transgenic lines were described earlier (Nakamura et al. 2008a, 2009). For imaging embryos, hatchlings or tissues were mounted with 1.2% low melting temperature agarose. Confocal pictures and image stacks were acquired using a Nikon C1 (eclipse Ti) confocal laser scanning microscope and the NIS elements AR software.

### Immunochemistry

Ovaries or testes from juvenile and adult fish were fixed with 4% paraformaldehyde/balanced salt solution (111 mM NaCl, 5.37 mM KCl, 1 mM CaCl_2_∙H_2_O, 0.6 mM MgSO_4_∙7H_2_O, 5 mM Hepes, pH 7.3) for 30 min on ice. After fixation samples were washed three times for 10 min with MABT buffer (100 mM maleic acid, 150 mM NaCl, pH 7.5, 0.1% Triton X-100) and subsequently twice for 30 min with MABDT buffer (MABT buffer complemented with 1% BSA and 1% DMSO). After blocking in MABDT-blocking buffer (MABDT buffer supplemented with 2% lamb or sheep serum), the tissues were incubated in MABDT-blocking buffer together with the primary antibody (1:150 dilution) overnight at 4 °C. Samples were then washed three times 5 min in MABDT buffer and washed again four times for 30 min in MABDT-blocking buffer on ice. Thereafter samples were incubated overnight at 4 °C with the secondary antibody diluted at 1:600 in MABDT-blocking buffer. Finally the tissues were washed in PBS, stained with Hoechst solution for 3 h at 4 °C, mounted, and imaged with a confocal microscope (Nikon C1 confocal microscope).

### In vivo Chromatin Immunoprecipitation

For in vivo chromatin immunoprecipitation, the EpiQuik Tissue Chromatin Immunoprecipitation kit (Epigentek) was used according to the manufacturer’s instructions, using 20 mg of testis tissue samples either from *dmrt1bY::GFP* or *deltadmrt1bY::GFP* transgenic fish (Hornung et al. 2007; Herpin et al. 2010) (20 testes for each) and GFP antibody (3 µg, Upstate) for immunoprecipitation. After tissue disaggregation and cell re-suspension, DNA was sheared by sonication (9 pulses of 10 s with an amplitude of 10%). After immunoprecipitation ([DKK1-(1/2) Fw01]: 5′-GATAACTCCGGCTGGGACGTTGAC-3′/[DKK–(1/2) Rv01]: 5′-ACAACACTGAAGTGCTACAGAAGTC-3′; [DKK1-(3) Fw02]: 5′-AGTATCAAGTGCTCAAGACGATCC-3′/[DKK1-(3) Rv02]: 5′-TACGAGCTGACATGTTCACATCTGCC-3′; [DKK1-(4/5) Fw03]: 5′-GCTGCAAGACAGGAAGAC-3′/[DKK1-(4/5) Rv03]: 5′-GTTAATAGTCATGCTCAGTCTG-3′; [R-spo1-(1) Fw01]: 5′-CATCGGATTTAACAGTTATGATTGC-3′/[R-spo1-(1) Rv01]: 5′-CGATAGTGATTGGTCAGTTA-3′; [R-spo1-(2/3) Fw02]: 5′-CATCGTGCCAACTTACAGCCAATC-3′/[R-spo1-(2/3) Rv02]: 5′-CTACCAAGACACGCTAGAAGCTCC; [R-spo1-(4) Fw03]: 5′-AAGTTGCTCAACACTTGTACAC-3′/[R-spo1-(4) Rv03]: 5′-AAGCAGAGACAATAGAATGCATC-3′; [R-spo1-(5) Fw04]: 5′-ATAAACATGTACAACAGTCATCTG-3′/[R-spo1-(5) Rv04]: 5′-TTCCACTCTCGGCAAGAAATCAG-3′; [HHIP-Fw01]: 5′-TAGAGTACGTCCGTCTACTG-3′/[HHIP-Rv01]: 5′-TGACAACAAAGTCGCAA-3′) primer sets were used for enrichment quantification by real-time PCR.

### Bioinformatic Analyses

Binding sites for Dmrt1bY were identified using the matrix provided by (Murphy et al. 2007) together with the Regulatory Sequence Analysis Tools portal; RSat (http://rsat.ulb.ac.be/rsat/, last accessed August 12, 2013).

### In Vitro Expression Regulation Analyses and Real-Time PCR

Medaka spermatogonial (SG3) and fibroblast-like (OLF) cell lines were cultured as described (Etoh 1988; Hong et al. 2004). For transfection cells were grown to 80% confluency in 6-well plates and transfected with 5 µg expression vector using FuGene (Roche) reagent as described by the manufacturer.

Total RNA was extracted from fish tissues or transfected cells using the TRIZOL reagent (Invitrogen) according to the supplier’s recommendation. After DNase treatment, reverse transcription was done with 2 µg total RNA using RevertAid First Strand Synthesis kit (Fermentas) and random primers. Real-time quantitative PCR was carried out with SYBR Green reagents and amplifications were detected with an i-Cycler (Biorad). All results are averages of at least two independent reverse transcription reactions. Error bars represent the standard deviation of the mean. Relative expression levels (according to the equation 2–DeltaCT) were calculated after correction of expression of elongation factor 1 alpha (ef1alpha) and brain expression was set to 1 as a reference.

## Supplementary Material

Supplementary figure S1 is available at *Molecular Biology and Evolution* online (http://www.mbe.oxfordjournals.org/).

## References

[mst130-B1] Bitgood MJ, Shen L, McMahon AP (1996). Sertoli cell signaling by Desert hedgehog regulates the male germline. Curr Biol..

[mst130-B2] Bourguiba S, Genissel C, Lambard S, Bouraima H, Carreau S (2003). Regulation of aromatase gene expression in Leydig cells and germ cells. J Steroid Biochem Mol Biol..

[mst130-B3] Bowles J, Feng CW, Spiller C, Davidson TL, Jackson A, Koopman P (2010). FGF9 suppresses meiosis and promotes male germ cell fate in mice. Dev Cell..

[mst130-B4] Canto P, Soderlund D, Reyes E, Mendez JP (2004). Mutations in the desert hedgehog (DHH) gene in patients with 46,XY complete pure gonadal dysgenesis. J Clin Endocrinol Metab..

[mst130-B5] Carmon KS, Gong X, Lin Q, Thomas A, Liu Q (2011). R-spondins function as ligands of the orphan receptors LGR4 and LGR5 to regulate Wnt/beta-catenin signaling. Proc Natl Acad Sci U S A..

[mst130-B6] Chassot AA, Gregoire EP, Magliano M, Lavery R, Chaboissier MC (2008). Genetics of ovarian differentiation: Rspo1, a major player. Sex Dev..

[mst130-B7] Chelly J, Concordet JP, Kaplan JC, Kahn A (1989). Illegitimate transcription: transcription of any gene in any cell type. Proc Natl Acad Sci U S A..

[mst130-B8] Chuang PT, McMahon AP (1999). Vertebrate Hedgehog signalling modulated by induction of a Hedgehog-binding protein. Nature.

[mst130-B9] Clark AM, Garland KK, Russell LD (2000). Desert hedgehog (Dhh) gene is required in the mouse testis for formation of adult-type Leydig cells and normal development of peritubular cells and seminiferous tubules. Biol Reprod..

[mst130-B10] Crisponi L, Deiana M, Loi A, (22 co-authors) (2001). The putative forkhead transcription factor FOXL2 is mutated in blepharophimosis/ptosis/epicanthus inversus syndrome. Nat Genet..

[mst130-B11] Cutting A, Chue J, Smith CA (2013). Just how conserved is vertebrate sex determination?. Dev Dyn..

[mst130-B12] De Felici M (2009). Primordial germ cell biology at the beginning of the XXI century. Int J Dev Biol..

[mst130-B13] Etoh (1988). Establishment and characterization of various cell lines from Medaka (Teleostei). Invertebrate and fish tissue culture.

[mst130-B14] Forconi M, Canapa A, Barucca M, (14 co-authors) (2013). Characterization of sex determination and sex differentiation genes in latimeria. PLoS One.

[mst130-B15] Franco HL, Yao HH (2012). Sex and hedgehog: roles of genes in the hedgehog signaling pathway in mammalian sexual differentiation. Chromosome Res..

[mst130-B16] Garcia-Ortiz JE, Pelosi E, Omari S, (12 co-authors) (2009). Foxl2 functions in sex determination and histogenesis throughout mouse ovary development. BMC Dev Biol..

[mst130-B17] Giraldo P, Montoliu L (2001). Size matters: use of YACs, BACs and PACs in transgenic animals. Transgenic Res..

[mst130-B18] Govoroun MS, Pannetier M, Pailhoux E, Cocquet J, Brillard JP, Couty I, Batellier F, Cotinot C (2004). Isolation of chicken homolog of the FOXL2 gene and comparison of its expression patterns with those of aromatase during ovarian development. Dev Dyn..

[mst130-B19] Graham P, Penn JK, Schedl P (2003). Masters change, slaves remain. Bioessays.

[mst130-B20] Guiguen Y, Fostier A, Piferrer F, Chang CF (2010). Ovarian aromatase and estrogens: a pivotal role for gonadal sex differentiation and sex change in fish. Gen Comp Endocrinol..

[mst130-B21] Haag ES, Doty AV (2005). Sex determination across evolution: connecting the dots. PLoS Biol..

[mst130-B22] Herpin A, Braasch I, Kraeussling M, Schmidt C, Thoma EC, Nakamura S, Tanaka M, Schartl M (2010). Transcriptional rewiring of the sex determining dmrt1 gene duplicate by transposable elements. PLoS Genet..

[mst130-B23] Herpin A, Nakamura S, Wagner TU, Tanaka M, Schartl M (2009). A highly conserved cis-regulatory motif directs differential gonadal synexpression of Dmrt1 transcripts during gonad development. Nucleic Acids Res..

[mst130-B24] Herpin A, Schartl M (2009). Molecular mechanisms of sex determination and evolution of the Y-chromosome: insights from the medakafish (Oryzias latipes). Mol Cell Endocrinol..

[mst130-B25] Herpin A, Schartl M (2008). Regulatory putsches create new ways of determining sexual development. EMBO Rep..

[mst130-B26] Herpin A, Schartl M (2011). Sex determination: switch and suppress. Curr Biol..

[mst130-B27] Herpin A, Schindler D, Kraiss A, Hornung U, Winkler C, Schartl M (2007). Inhibition of primordial germ cell proliferation by the medaka male determining gene Dmrt I bY. BMC Dev Biol..

[mst130-B28] Hong Y, Liu T, Zhao H, Xu H, Wang W, Liu R, Chen T, Deng J, Gui J (2004). Establishment of a normal medakafish spermatogonial cell line capable of sperm production in vitro. Proc Natl Acad Sci U S A..

[mst130-B29] Hooper JE, Scott MP (2005). Communicating with Hedgehogs. Nat Rev Mol Cell Biol..

[mst130-B30] Hornung U, Herpin A, Schartl M (2007). Expression of the male determining gene dmrt1bY and its autosomal coorthologue dmrt1a in medaka. Sex Dev..

[mst130-B31] Hudson QJ, Smith CA, Sinclair AH (2005). Aromatase inhibition reduces expression of FOXL2 in the embryonic chicken ovary. Dev Dyn..

[mst130-B32] Ingham PW, McMahon AP (2001). Hedgehog signaling in animal development: paradigms and principles. Genes Dev..

[mst130-B33] Kashimada K, Pelosi E, Chen H, Schlessinger D, Wilhelm D, Koopman P (2011). FOXL2 and BMP2 act cooperatively to regulate follistatin gene expression during ovarian development. Endocrinology.

[mst130-B34] Kim Y, Kobayashi A, Sekido R, DiNapoli L, Brennan J, Chaboissier MC, Poulat F, Behringer RR, Lovell-Badge R, Capel B (2006). Fgf9 and Wnt4 act as antagonistic signals to regulate mammalian sex determination. PLoS Biol..

[mst130-B35] Kobayashi T, Matsuda M, Kajiura-Kobayashi H, Suzuki A, Saito N, Nakamoto M, Shibata N, Nagahama Y (2004). Two DM domain genes, DMY and DMRT1, involved in testicular differentiation and development in the medaka, Oryzias latipes. Dev Dyn..

[mst130-B36] Koopman P, Gubbay J, Vivian N, Goodfellow P, Lovell-Badge R (1991). Male development of chromosomally female mice transgenic for Sry. Nature.

[mst130-B37] Krentz AD, Murphy MW, Sarver AL, Griswold MD, Bardwell VJ, Zarkower D (2011). DMRT1 promotes oogenesis by transcriptional activation of Stra8 in the mammalian fetal ovary. Dev Biol..

[mst130-B38] Liu CF, Liu C, Yao HH (2010). Building pathways for ovary organogenesis in the mouse embryo. Curr Top Dev Biol..

[mst130-B39] Masuyama H, Yamada M, Kamei Y, Fujiwara-Ishikawa T, Todo T, Nagahama Y, Matsuda M (2012). Dmrt1 mutation causes a male-to-female sex reversal after the sex determination by Dmy in the medaka. Chromosome Res..

[mst130-B40] Matson CK, Murphy MW, Sarver AL, Griswold MD, Bardwell VJ, Zarkower D (2011). DMRT1 prevents female reprogramming in the postnatal mammalian testis. Nature.

[mst130-B41] Matsuda M (2005). Sex determination in the teleost medaka, Oryzias latipes. Annu Rev Genet..

[mst130-B42] Matsuda M, Nagahama Y, Shinomiya A, (13 co-authors) (2002). DMY is a Y-specific DM-domain gene required for male development in the medaka fish. Nature.

[mst130-B43] McLaren A, Southee D (1997). Entry of mouse embryonic germ cells into meiosis. Dev Biol..

[mst130-B44] McLeod JF, Cooke NE (1989). The vitamin D-binding protein, alpha-fetoprotein, albumin multigene family: detection of transcripts in multiple tissues. J Biol Chem..

[mst130-B45] Meinhardt A, O'Bryan MK, McFarlane JR, Loveland KL, Mallidis C, Foulds LM, Phillips DJ, de Kretser DM (1998). Localization of follistatin in the rat testis. J Reprod Fertil..

[mst130-B46] Menke DB, Page DC (2002). Sexually dimorphic gene expression in the developing mouse gonad. Gene Expr Patterns..

[mst130-B47] Murphy MW, Zarkower D, Bardwell VJ (2007). Vertebrate DM domain proteins bind similar DNA sequences and can heterodimerize on DNA. BMC Mol Biol..

[mst130-B48] Nakamoto M, Matsuda M, Wang DS, Nagahama Y, Shibata N (2006). Molecular cloning and analysis of gonadal expression of Foxl2 in the medaka, Oryzias latipes. Biochem Biophys Res Commun..

[mst130-B49] Nakamura S, Aoki Y, Saito D, Kuroki Y, Fujiyama A, Naruse K, Tanaka M (2008a). Sox9b/sox9a2-EGFP transgenic medaka reveals the morphological reorganization of the gonads and a common precursor of both the female and male supporting cells. Mol Reprod Dev..

[mst130-B50] Nakamura S, Kobayashi K, Nishimura T, Higashijima S, Tanaka M (2010). Identification of germline stem cells in the ovary of the teleost medaka. Science.

[mst130-B51] Nakamura S, Kurokawa H, Asakawa S, Shimizu N, Tanaka M (2009). Two distinct types of theca cells in the medaka gonad: germ cell-dependent maintenance of cyp19a1-expressing theca cells. Dev Dyn..

[mst130-B52] Nakamura S, Saito D, Tanaka M (2008b). Generation of transgenic medaka using modified bacterial artificial chromosome. Dev Growth Differ..

[mst130-B53] Nakamura S, Watakabe I, Nishimura T, Toyoda A, Taniguchi Y, Tanaka M (2012). Analysis of medaka sox9 orthologue reveals a conserved role in germ cell maintenance. PLoS One.

[mst130-B54] Nanda I, Kondo M, Hornung U, (12 co-authors) (2002). A duplicated copy of DMRT1 in the sex-determining region of the Y chromosome of the medaka, Oryzias latipes. Proc Natl Acad Sci U S A..

[mst130-B55] Nitta H, Osawa Y, Bahr JM (1991). Multiple steroidogenic cell populations in the thecal layer of preovulatory follicles of the chicken ovary. Endocrinology.

[mst130-B56] O'Hara WA, Azar WJ, Behringer RR, Renfree MB, Pask AJ (2011). Desert hedgehog is a mammal-specific gene expressed during testicular and ovarian development in a marsupial. BMC Dev Biol..

[mst130-B57] Ottolenghi C, Pelosi E, Tran J, Colombino M, Douglass E, Nedorezov T, Cao A, Forabosco A, Schlessinger D (2007). Loss of Wnt4 and Foxl2 leads to female-to-male sex reversal extending to germ cells. Hum Mol Genet..

[mst130-B58] Pannetier M, Fabre S, Batista F, Kocer A, Renault L, Jolivet G, Mandon-Pepin B, Cotinot C, Veitia R, Pailhoux E (2006). FOXL2 activates P450 aromatase gene transcription: towards a better characterization of the early steps of mammalian ovarian development. J Mol Endocrinol..

[mst130-B59] Parma P, Radi O, Vidal V, Chaboissier MC, Dellambra E, Valentini S, Guerra L, Schedl A, Camerino G (2006). R-spondin1 is essential in sex determination, skin differentiation and malignancy. Nat Genet..

[mst130-B60] Pierucci-Alves F, Clark AM, Russell LD (2001). A developmental study of the Desert hedgehog-null mouse testis. Biol Reprod..

[mst130-B61] Pisarska MD, Bae J, Klein C, Hsueh AJ (2004). Forkhead l2 is expressed in the ovary and represses the promoter activity of the steroidogenic acute regulatory gene. Endocrinology.

[mst130-B62] Schmidt D, Ovitt CE, Anlag K, Fehsenfeld S, Gredsted L, Treier AC, Treier M (2004). The murine winged-helix transcription factor Foxl2 is required for granulosa cell differentiation and ovary maintenance. Development.

[mst130-B63] Sekido R, Lovell-Badge R (2008). Sex determination involves synergistic action of SRY and SF1 on a specific Sox9 enhancer. Nature.

[mst130-B64] Shinomiya A, Shibata N, Sakaizumi M, Hamaguchi S (2002). Sex reversal of genetic females (XX) induced by the transplantation of XY somatic cells in the medaka, Oryzias latipes. Int J Dev Biol..

[mst130-B65] Siegfried KR (2010). In search of determinants: gene expression during gonadal sex differentiation. J Fish Biol..

[mst130-B66] Smith CA, Shoemaker CM, Roeszler KN, Queen J, Crews D, Sinclair AH (2008). Cloning and expression of R-Spondin1 in different vertebrates suggests a conserved role in ovarian development. BMC Dev Biol..

[mst130-B67] Spicer LJ, Sudo S, Aad PY, Wang LS, Chun SY, Ben-Shlomo I, Klein C, Hsueh AJ (2009). The hedgehog-patched signaling pathway and function in the mammalian ovary: a novel role for hedgehog proteins in stimulating proliferation and steroidogenesis of theca cells. Reproduction.

[mst130-B68] Suster ML, Abe G, Schouw A, Kawakami K (2011). Transposon-mediated BAC transgenesis in zebrafish. Nat Protoc..

[mst130-B69] Szczepny A, Hime GR, Loveland KL (2006). Expression of hedgehog signalling components in adult mouse testis. Dev Dyn..

[mst130-B70] Tisdall DJ, Hudson N, Smith P, McNatty KP (1994). Localization of ovine follistatin and alpha and beta A inhibin mRNA in the sheep ovary during the oestrous cycle. J Mol Endocrinol..

[mst130-B71] Tomizuka K, Horikoshi K, Kitada R, (12 co-authors) (2008). R-spondin1 plays an essential role in ovarian development through positively regulating Wnt-4 signaling. Hum Mol Genet..

[mst130-B72] Uhlenhaut NH, Jakob S, Anlag K, (15 co-authors) (2009). Somatic sex reprogramming of adult ovaries to testes by FOXL2 ablation. Cell.

[mst130-B73] Wang DS, Kobayashi T, Zhou LY, Paul-Prasanth B, Ijiri S, Sakai F, Okubo K, Morohashi K, Nagahama Y (2007). Foxl2 up-regulates aromatase gene transcription in a female-specific manner by binding to the promoter as well as interacting with ad4 binding protein/steroidogenic factor 1. Mol Endocrinol..

[mst130-B74] Wilhelm D, Palmer S, Koopman P (2007). Sex determination and gonadal development in mammals. Physiol Rev..

[mst130-B75] Wilkins AS (2007). Between “design” and “bricolage": genetic networks, levels of selection, and adaptive evolution. Proc Natl Acad Sci U S A..

[mst130-B76] Wittbrodt J, Shima A, Schartl M (2002). Medaka—a model organism from the far East. Nat Rev Genet..

[mst130-B77] Yao HH (2005). The pathway to femaleness: current knowledge on embryonic development of the ovary. Mol Cell Endocrinol..

[mst130-B78] Yao HH, Matzuk MM, Jorgez CJ, Menke DB, Page DC, Swain A, Capel B (2004). Follistatin operates downstream of Wnt4 in mammalian ovary organogenesis. Dev Dyn..

[mst130-B79] Zhang Y, Li F, Sun D, Liu J, Liu N, Yu Q (2011). Molecular analysis shows differential expression of R-spondin1 in zebrafish (*Danio rerio*) gonads. Mol Biol Rep..

